# Historical Review of the Progress of Dental Surgery in the United States

**Published:** 1851-10

**Authors:** 


### ARTICLE VIII.

### *Historical Review of the Progress of Dental Surgery in the United States, with Reflections upon the Causes that have Accelerated it, and the means Necessary for its further Advancement.*

In every department of human industry, the history of the United States shows a progress and development incomparably greater than have before been exhibited by any nation. The circumstances of this country are entirely novel in the experience of the world, and in every particular, the distinctive element has been exceedingly favorable to progress. Immense extent of fertile territory invites multitudes from over-populated and misgoverned regions, to become proprietors of a soil reserved for them from the foundation of the world, and waiting only for occupation to pour out its long accumulated riches. Political institutions, as free and equal as the heart could wish or the mind desire, draw together, under their generous banner, the noble-hearted and bold spirited from all lands. Universal education develops the effective powers and stimulates the energetic passions of the common mind. A wonderful commerce quickens the impulses of the people by allying their interests to the interests of all lands, extending a network of acute sensibilities over the whole globe, and making this country the sensorium commune of the world.

Before, under circumstances most favorable to human development, there was always some fetter upon the mind; something jealously guarded from the inquiry of the curious; something true by arbitrary decree, or false by the force of grim authority. Religion, of all subjects, the most interesting and exciting to the mind, the most ennobling to the heart, the most invigorating to the understanding, had been almost invariably a forbidden field to the bold inquirer. Politics, the religion of this world, next to the business of eternity, the most important of all themes, had also, except upon a few fitful opportunities, been an unlawful subject for examination. Shut out by authority from the most interesting pursuits pertaining to time and eternity, the restless mind was driven into oblique lines of thought—was forced to weary powers it could not employ—fatigue desires it could not satisfy. Under such circumstances, rapid development was impossible. As in a garden of the olden time, the business of the gardener was to trim and prune his plants until every dwarfish tree should present a regular form as far different as possible from that designed by nature. So, the governing institutions of the old world had only in view to check the growth of mind, and to train it to obedience to artificial forms.

In this country, all was changed. A multitude of ardent minds found themselves suddenly let loose upon all subjects, for the first time, unprotected. Each was at full liberty to select its object, choose its mode of acquisition and carry to the utmost its attainment.

That scenes and results new and startling must result from causes themselves so novel and efficient might easily have been foreseen. That evils, or what had heretofore been considered such, must be developed with terrible exuberance and in gigantic magnitude could readily have been predicted ; but that good, of a quality heretofore unknown, in quantity not conceived of, and of universality as the capacity of men to receive it, must be the grand ultimate consequence is as certain as that man was fitted by his Creator to subdue the world around him and within him, to the necessities of his own happiness. From the nature of things, the active in such a country must predominate over the contemplative. The world around is too vast, and the immediate and pressing occupations too many to permit quiet excursions into the invisible realms of the ideal world, except as a holiday trip. Physical science has made great progress ; but in art, or the application of the truths of science to practical good, the onward movement has been with far greater velocity. .

Men are always willing to pay for comfort to the very utmost extent of their means. In this country, the masses are able to pay for them. Hence the ready way to wealth is to increase means and appliances by which the wants of the many are supplied, their forces multiplied, their labors diminished, or their infirmities relieved or counteracted. Here, it is the multitude who pay, and the multitude look for comfort as the great desideratum. Where it is otherwise, the efforts of ingenuity must be directed to gratify the luxurious few, who, possessing comfort, are ready to lavish their surplus means upon the gratification of cultivated or capricious fancy, or what is commonly known as taste. Under such circumstances, excellence of execution, which creates a variety in the possession, takes the place of the universal adaptedness which anticipates a universal demand.

Among the arts medicine has always been prominent. Founded upon a universal want, it has always been of universal acceptability.

In the progress of the arts in the United States, medicine has received a large share of attention and made very considerable advancement; but, certainly, its progress will not bear comparison with that made by the more purely physical arts. There is no contrast between past and present medicine at all corresponding to the difference between an antiquated flat-boat and the modern leviathan steamer; between the former post-chase and the modern locomotive.

Superficial observers, regarding this disproportionate development, have been led to complain, that medicine has been laggard in the movement of mind; but we must consider that medicine had reached great maturity when mechanics were in infancy. Medicine has always been cultivated; has always been a science, and had acquired at the time the new movement took place, by far the greater part of what was possible for it to acquire. Moreover, medicine, from its nature, is restricted in its bounds, and limited in its means. It is a struggle against what is ultimately inevitable, by instrumentalities only partially effective.

Yet, *practical* medicine has made much advancement among us; and whatever may be thought of the great excellence attained by foreigners in the knowledge of the minute animal structure and the discrimination of morbid conditions and locations, we have no hesitation in asserting, that the proper art of cure has been brought to as great perfection in America and through the efforts of Americans, as it has been elsewhere and by other agents.

In surgery, the artistic talent of the American mind has had considerable scope for action, and many valuable improvements have been made by our surgeons, of whom we have never been without some acknowledged equal to the very best of their contemporaries. Indeed, when we consider that our operators have seldom had such abundant opportunities for the exercise of their skill, as have been afforded to those of other nations by the terrible accidents of war, it is surprising that our surgery has been able to sustain its relative position. Yet, in all its departments, it will suffer nothing in comparison with the highest development of the art yet attained in Europe. Dental surgery, as at present practiced, is almost an American creation, for although operations upon the teeth have been practiced since the days of the Pharaohs, and probably before, yet the rude and simple character of the early manipulations hardly give them a claim to be regarded among the effects of scientific art, and until comparatively lately, but very little improvement seems to have been made in this department of surgery.

To the earlier physiologists, the teeth were an inscrutable mystery, and their diseases were as unintelligible as their structure. Hippocrates regarded them as a glutinous exudation from the jaws. Aristotle taught that men had more teeth than women, an unpardonable error, because so easily corrected ; he also supposed, that these organs continued to grow during life. Aretæus says, that God only knows the cause of tooth-ache : and Celsus recommends as odontalgic applications, the actual cautery and hot oil.

After the writings of Ambrose Paré had given a new impulse and a right direction to surgery, dentistry began to share the interest awakened for operative medicine. The organization of the teeth were considered in the several works on physiology, and was at length almost truthfully described by Leeuwenhœk Yet when Fauchard published in 1728 the first systematic treatise on dental surgery, operative dentistry was evidently in a rude state. From this time, however, considerable improvement was made, up to the close of the century, when Hunter wrote his famous work on the teeth, and thus constituted another epoch in the history of the art. Bichat and other French anatomists and physiologists also paid a fair proportion of attention to the teeth, and a number of surgeons and dentists published books and papers upon the subject. In short, dentistry had now become an important and valued branch of the healing, and perhaps we may add, the decorative, art.

In the early years of our colonial existence, it is probable that no attention was paid to dentistry. The people were scattered over a wide extent of country, and generally could have had no money to expend in such extravagant luxuries as dental operations, even if such had been at their option. Their teeth had probably not yet formed the American habit of premature decay, and if occasionally one became refractory, it is probable the village blacksmith removed it with his pincers.

But as wealth increased and cities became populous, arts of all kinds became in better demand, and what previously were unknown or regarded as luxuries, became recognized wants.

The first immigrant who ventured to practice dentistry as an exclusive pursuit, seems to have been a Mr. Woofendale, who settled in New York in 1766. Not meeting with success, he returned to Europe two years afterwards. During the revolution, M. Le Mair, came over with the French army, and established himself as a dentist, and was followed by Whitelock an Englishman. Of these persons, Dr. Harris observes "with regard to the professional abilities of Le Mair and Whitelock, little is known, but it is probable they were limited and that their practice consisted chiefly in constructing artificial teeth from blocks of ivory."

Mr. Gardette, so long and so favorably known to the citizens of Philadelphia, came to this country in 1783, and with the exception of Mr. John Greenwood, may be regarded as the first dentist worthy of the name, who established himself in this country.

Mr. Greenwood was an American by birth, and commenced practice in New York, in 1788, where he continued almost the only dental surgeon for twelve years—a fact which shows how very little dentistry was appreciated in those days.

Mr. Greenwood was an industrious and ingenious man, and soon acquired reputation among the few who perceived the value of such services as the then state of the art enabled him to render.

An upper set of teeth, carved from hippopotamus ivory, is yet to be seen in the museum of the Baltimore College of Dental Surgery, made by Mr. Greenwood, for General Washington, together with a letter from General Washington to Mr. Greenwood, expressing his gratification with his services. The teeth were doubtless good of their kind, and for their time, but by the side of a set of modern fashion, they may be supposed to have been intended for some different purpose.

Dr. Hudson, of Dublin, established himself in Philadelphia, in 1805, where he acquired, and deservedly too, a great reputation which he enjoyed for twenty years. We are not aware that any of these gentlemen contributed any thing to the literature of their profession.* The constant demand for their services by persons able to remunerate them, called forth all their ingenuity in devising the readiest and most complete substitutes for deficiencies of the natural teeth, and made them skillful in performing the operations then in use upon these organs when diseased. Carving artificial teeth from bone, neatly introducing human teeth, extracting and filling with more or less completeness, and constructing the cumbrous spring-work by which their rudely constructed sets were made to do their reluctant office—these occupied the attention of the dentist. He had no help from without. Himself designer and artificer, he was compelled to rely upon his own mechanical skill for whatever he needed in his manipulations. Even his instruments were of his own workmanship.

* Dr. Gardette wrote a paper on the Transplantation of Teeth, recently republished in the American Journal of Dental Science.—Eds.

Under such circumstances, there was doubtless much improvement made in mechanical and operative dentistry, though the condition of the profession did not create a demand for literary effort. But the gentlemen above named did not restrict themselves to the mere mechanism of the profession. They were well informed in general medicine, and their pupils were required to attend medical lectures, and read medical books, and thus a class of educated dentists was gradually formed.

This class, however, was necessarily very small. Men engaged in a lucrative practice, operating during the day, and doing goldsmith's work at night, could not devote much time to the instruction of pupils. Besides, the advantage of a certificate of pupilship from one of the highly reputed dentists, was so great that very large fees were paid for such tuition, imperfect as it was. Five hundred dollars was commonly asked for the office fee of a student, and of course, very few could avail themselves of such dearly purchased advantages.

The great benefit conferred by these men upon the profession, independently of what improvements in operating they may have made, consisted in establishing a class of educated gentlemen, acquainted with medicine, but practicing dentistry as a specialty. In fact, they laid the foundation of the present *respectability* of dentistry.

In 1800, Dr. Horace Hayden commenced business in Baltimore. He had enjoyed no instruction except what he had derived from the few books then to be found, and no doubt was very ill qualified to practice. But he was a very honest minded, enthusiastic man, and learned as fast as he could, and from every accessible source. He studied medicine and surgery thoroughly, and became expert in geological science, and well versed in natural history. His bent of mind was decidedly *scientific*, and his untiring curiosity penetrated into every open door of nature.

Dr. Hayden was not only a student, he was a good writer, but never wrote unless he had something to say. His writings, though chiefly upon medicine and natural science, include some valuable papers upon dental subjects. In 1825, he published in the New York Medical Recorder, a paper upon Conjoined Suppuration of the Gums. A year or two previously, had appeared a paper by him, on the nature, growth and formation of the human teeth, with an explanation of the cause of their decay, particularly of its uncommon prevalence in the middle and northern states of North America—a paper valuable, as showing the existence at that time of the peculiarity so distressingly observable, and so unaccountable now—and also the acute and observing character of the writer's mind. Another paper upon the appearance of the teeth of those who have died from strangulation, was published in the Encyclopedia of Medicine, and another in the New York Repository, on the use and functions of the salivary lachrymal, and other glands in the human system. This last paper was published in 1818, and prior to all other publications upon dental subjects, in our medical journals, at least so far as we are informed.

Dr. Hayden did much to elevate the literary and scientific standing of the dental profession. He was the first to give it caste in Baltimore. His own associations were with the most learned and literary men of the city, and necessarily his personal consideration was reflected upon his profession.

Books and papers on dental surgery and collateral subjects, now began to multiply. Mr. L. S. Parmly, published his "Practical Guide," in 1819. Dr. E. Parmly and Dr. Flagg, appeared as authors in 1822. Dr. Trenor in 1828, and Dr. Fitch's large and valuable work was published in 1829. In 1839 Dr. Harris brought out his well known and excellent work on practice, and many smaller works, by various authors, have, from time to time, been given to the public.

In periodical literature, nothing was attempted until the publication of the American Journal and Library of Dental Science, in 1839. The number of dentists was now sufficient to sustain such a publication, and its effect in consolidating and vivifying the scattered elements of the profession was as cheering as remarkable. The bold step of founding a college for the regular education of dentists, soon followed, and the institution at Baltimore was ushered into existence. Then followed the American Society of Dental Surgeons, the several state associations, the Ohio College, and a number of additional periodicals.

While these aggregated efforts were making to consolidate and legitimatize the profession of the dentist great improvements had been made in operative and mechanical dentistry. The manufacture of artificial teeth has been brought to such perfection, that it seems impossible to make any material improvement in them. The mode of inserting artificial pieces, has been greatly improved—filling is now done with far greater completeness and certainty than before ; dental instruments have been much improved in form, adaptedness and construction, and the *medicine* of the art, the indispensable collateral knowledge of the body, its diseases and their remedies, has been much advanced.

The progress of dentistry for the last twenty years, has indeed been prodigious, and American dentistry now stands foremost in the world.

The causes of this progress must be sought, first in the melancholy fact that the teeth of Americans are subject to decay to an almost incredible extent. We all know that a perfect set of teeth is now a curiosity. That scarcely a lady out of her teens can be met with whose mouth is not made up of plugs and porcelain, that the dentist's bill is larger than the grocer's, and that tooth-ache, under our free institutions is so universally tormenting, as fully to countervail the advantage of trial by jury—we know these things, and inasmuch as demand regulates supply and stimulates exertion, there is no difficulty in accounting for the improvement in an art so much in use as dentistry.

In the next place we must look for the cause of progress in the facts we have already considered as constituting the philosophy of our national character. The universality of want is felt by the *moneyed multitude*, and not by the *moneyed few*. Hence the direction of skill must be to supply the wants of the many, rather than the tastes of the few. Dentistry has not only a brisk demand, but a good market. And again, we must look for the cause of progress of dentistry in the freedom to think and to act, and the facility of communicating thought and action so distinctive of our race and times.

(To be Continued.)

